# Reliability and Validity of an Ultrasound-Derived Measure for Axial Patellofemoral Alignment

**DOI:** 10.1177/23259671241281362

**Published:** 2024-10-11

**Authors:** Elliot M. Greenberg, Megan Barnes, J. Todd Lawrence, Naomi Brown, Brendan Williams

**Affiliations:** * Children’s Hospital of Philadelphia Sports Medicine and Performance Center, Philadelphia, Pennsylvania, USA; † Department of Orthopaedic Surgery, Perelman School of Medicine, University of Pennsylvania, Philadelphia, Pennsylvania, USA; ‡ Department of Pediatrics, Perelman School of Medicine, University of Pennsylvania, Philadelphia, Pennsylvania, USA; Investigation performed at Children's Hospital of Philadelphia, Philadelphia, Pennsylvania, USA

**Keywords:** adolescent, musculoskeletal ultrasound, patellar dislocation, patellar subluxation, pediatric

## Abstract

**Background:**

Axial extensor mechanism alignment is routinely assessed in patients with patellofemoral instability.Although many of these assessments are described using magnetic resonance imaging, it is plausible that ultrasound (US) imaging could be utilized to provide similar information in a more cost-effective and time-efficient manner.

**Purpose:**

To (1) describe and assess the reliability of a novel measure of extensor mechanism alignment of the patellofemoral joint using musculoskeletal US and (2) establish the construct validity of this measure through comparison of patients with and without patellar instability.

**Study Design:**

Cohort study (diagnosis); Level of evidence, 3.

**Methods:**

Patients with (n = 24; 14.2 ± 3.1 years; 83% female) and without (n = 26; 14.7 ± 2.8 years; 69% female) a clinical history of patellofemoral instability (PFI) participated. Extensor mechanism alignment was assessed bilaterally on US, measuring the distance between the midpoint of the patellar tendon to the lateral trochlear ridge (MPT-LTR). Interrater reliability of the measurements was assessed using the intraclass correlation coefficient (ICC), with a minimum of 1 week between measurements for test-retest reliability. Differences between limbs were assessed using paired-samples *t* tests, and between-group differences were compared using independent-samples *t* tests.

**Results:**

Patients with PFI demonstrated a significantly smaller MPT-LTR distance than healthy controls on both their involved (8.1 ± 3.6 vs 12.6 ± 2.6 mm) and uninvolved (8.9 ± 3.4 vs 12.9 ± 2.4 mm) limbs (*P* < .001 for both), indicating greater lateralization of the patellar tendon relative to the trochlea. There were no differences found between limbs for either patients with PFI (*P* = .26) or controls (*P* = .46). Interrater reliability was good (ICC = 0.785; 95% confidence interval [CI], 0.579-0.890), and test-retest reliability (n = 8) was excellent (ICC = 0.958; 95% CI, 0.790-0.992).

**Conclusion:**

The US-based MPT-LTR distance demonstrated good-to-excellent reliability. When compared with controls, the MPT-LTR distance was smaller in patients with PFI, indicating greater lateralization of the extensor mechanism.

Patellofemoral instability (PFI) is a common condition among pediatric and adolescent athletes with an incidence in 10- to 17-year-olds that is 6 times higher than that of adults.^17^ There is a high risk of recurrent instability in this population, with studies demonstrating repeat dislocations in 30% to 54% of cases.^8,13^ The high rate of repeat instability may be rooted in the multifactorial nature of the pathophysiology involved in patellar instability. The stability of the patellofemoral joint is a complex interplay between osseus structures, soft tissue restraints, dynamic muscular stabilizers, and lower limb biomechanics. Instability occurs when the net force of laterally directed forces encountered by the patella overwhelm the capacity of these restraining structures and the patella moves outside of the lateral trochlear ridge. The relative alignment of the tibial tubercle to the trochlear groove (TT-TG) is a widely studied anatomic risk factor, with the understanding that a larger degree of lateralization of the tubercle relative to the trochlea may increase the lateral force vector encountered by the patellofemoral joint.^15^ However, recent literature suggests that other measures quantifying the axial relationship of the extensor mechanism and the patellofemoral joint may demonstrate better diagnostic accuracy than the TT-TG distance.^9,16^

Over the past decade, there has been growing interest and increased use of point-of-care ultrasound (US) in orthopaedics to assist with differential diagnosis and clinical decision-making.^10,12,14^ Musculoskeletal US offers real-time visualization of anatomy with improved efficiency, decreased patient burden, and cost associated with other imaging modalities such as traditional radiographs or magnetic resonance imaging (MRI).^10^ While US is unable to visualize deep anatomic structures or penetrate bone, the superficial nature of the patellofemoral joint makes it an easily accessible target for the use of US. Previous research has described use of US for patellofemoral alignment; none of these studies have focused on those with patellar instability, and the methodology employed does not fully consider the axial alignment of the patellofemoral joint, limiting its direct application to this population.^1-3,6,7^

The purpose of this study was to describe and assess the reliability of a novel measure of patellotrochlear alignment using musculoskeletal US. We secondarily sought to establish construct validity of this measure through comparison of patients with versus without patellar instability. We hypothesized that subjects with a history of PFI or dislocation would have a more laterally positioned patellar tendon when compared with healthy controls.

## Methods

### Participant Recruitment

Patients 8 to 21 years of age with and without a clinical history of PFI were recruited from a single institution. Before enrollment, the patient and/or parent filled out a brief electronic questionnaire that included demographic information, sports activities, and injury history to evaluate for inclusion and exclusion criteria of the study. Inclusion criteria for the PFI cohort required the patient to have at least 1 patellar subluxation or dislocation event that was diagnosed by a fellowship-trained, pediatric orthopaedic sports medicine specialist or pediatric orthopaedic surgeon. This diagnostic process was not controlled as part of the study design and was based upon history, imaging, and physical examination procedures as part of standard medical or diagnostic procedures. After diagnosis by the orthopaedic provider, patients were approached by a member of the study team to determine eligibility for the additional inclusion criterion (ability to sit comfortably with the knee in full extension) and determine parent/patient interest in participating in the study. Data collection occurred at a later time mutually convenient for both patient and examiner. The time from dislocation event to US assessment was not controlled.

The control group consisted of patients who were evaluated for nonknee issues at our institution and who had no history of knee-related issues. Participants were excluded from both groups if they had any history of knee surgery or if they had any pre-existing development disorder or previous knee injury that may have affected patellar or trochlear morphology (eg, patellar fracture). The protocol for this study received institutional review board approval, and parent and patient consent and/or patient assent were obtained before the initiation of any study procedures.

### US Measurement Procedures

#### Patient Positioning

**Figure 1.**
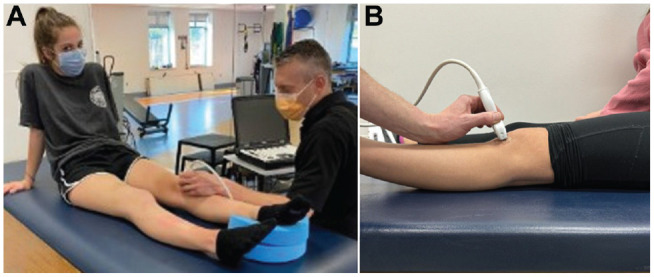
(A) Patient positioning for US measurement. (B) Lateral view showing probe orientation. US, ultrasound.

All images were obtained using a GE LOGIQ US unit and a 4-cm linear transducer. Subjects were positioned in a long sitting position with the knee relaxed and in full extension. A foam donut was placed under each subject's heel to stabilize the limb, keeping the limb in a neutral position (defined as foot in vertical position), and limit any rotational movement during the procedure (Figure 1A).

#### Imaging Acquisition

Axial plane patellofemoral alignment was assessed via US in the following manner. The US probe was placed on the anterior aspect of the subject's knee and angled slightly superiorly to allow for visualization of the patellar tendon and trochlear groove within a single view. To standardize the viewing window of the patellofemoral articulation, the examiner first identified the inferior pole of the patella and then moved the probe inferiorly to the point at which the most proximal aspect of the patellar tendon was in full view. With the patellar tendon in view, the probe was oriented to also capture the trochlear groove in the same image (Figure 1B). Images were then captured and transferred to the hospital's Picture Archiving and Communication System (PACS) for measurement. The same procedure was performed twice for each limb, and the mean of the 2 measures was utilized for data analysis. The order of limb imaging was randomized by asking the subject which limb they preferred to measure first.

#### Measurement Procedure

**Figure 2.**
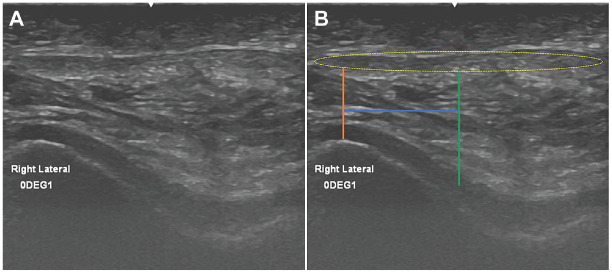
(A) Axial ultrasound slice of a right knee. (B) Width of the patellar tendon (yellow dotted oval) was measured, and the MPT (green line) was identified; the peak of the LTR was then identified (orange line). The linear distance between these 2 lines (blue line) was the MPT-LTR distance; a smaller distance indicated a more lateral patellar tendon position. MPT, midpoint of the patellar tendon; LTR, lateral trochlear ridge.

The following measurement procedure was utilized to determine the linear distance from the midpoint of the patellar tendon (MPT) to the lateral trochlear ridge (LTR). The medial-lateral width of the patellar tendon in the axial plane was first measured, and a perpendicular line was then drawn at the midpoint of the patellar tendon. The peak of the lateral trochlear ridge was defined operationally as the apex of the curve, and a perpendicular line was drawn to bisect this point. The distance between the MPT and LTR was then determined (Figure 2). In this measurement procedure, a smaller MPT-LTR value indicates a more laterally oriented patellar tendon position relative to the trochlea.

All data-collection procedures and postprocessing measurements were performed independently by 2 investigators (E.G. and M.B.). Investigator 1 (E.G.) had approximately 6 years of US experience with some formal training in continuing education settings, while investigator 2 (M.B.) had <6 months of US experience before engaging in this study. The investigators were blinded to each other's measurements at all times.

To assess intrarater reliability, a second set of measurements were conducted on a convenience sample of subjects based upon patient availability. The second measures were performed by the same examiner who collected the initial data, a minimum of 1 week from the initial measurement. This time duration was selected to allow a sufficient washout period and limit any potential recall bias.

### Statistical Analysis

The intra- and interrater reliability of the US measurements were assessed using the intraclass correlation coefficient (ICC) with 95% confidence interval (CI). Minimal detectable change (MDC), the minimal amount of change in in the measurement that must occur to be sure that the change is not simply attributable to measurement error, was calculated using the formula MDC = standard error of measurement × 1.96 × √2. Differences in the MPT-LTR distance between the involved and uninvolved limbs were assessed with the paired-samples *t* test, while differences between the PFI and control groups were analyzed using the independent-samples Student *t* test. All analyses were performed using SPSS (Version 27.0; IBM), and an a priori significance level of *P* < .05 was utilized to determine significance.

## Results

A total of 50 participants who met the study criteria were included; there were 24 patients with PFI (mean age, 14.2 ± 3.1 years; 83% female) and 26 controls (mean age, 14.7 ± 2.8 years; 69% female). There were no significant differences in baseline characteristics between groups. Full demographic information is available in Table 1.

**Table 1:** Patient Characteristics According to Study Group (N = 50)^*a*^

	PFI (n = 24)	Control (n = 26)	*P*
Age, y	14.2 ± 3.1	14.7 ± 2.8	.513
Height, cm	161.5 ± 14.3	161.9 ± 9.9	.845
Weight, kg	55.2 ± 14.5	57.7 ± 12.7	.467
Sex, female	20 (83%)	18 (69%)	.308

a Data are presented as mean ± SD or n (%). PFI, patellofemoral instability.

The PFI group demonstrated significantly smaller MPT-LTR distances than the control group on both the involved (8.1 ± 3.6 vs 12.6 ± 2.6 mm) and uninvolved (8.9 ± 3.4 vs 12.9 ± 2.4 mm) limb (*P* < .001 for both), indicating greater extensor mechanism lateralization relative to the trochlea (Table 2). The mean differences between the PFI and control groups were 4.5 and 4.0 mm in the involved and uninvolved limbs, respectively. No significant differences were identified on between-limb comparisons in either the PFI group (mean difference, 0.9 mm; 95% CI, 0.7-2.5 mm; *P* = .263) or control group (mean difference, 0.4 mm; 95% CI, 0.7-1.6 mm; *P* = .459).

**Table 2:** MPT-LTR Distance According to Study Group^*a*^

Side	PFI	Control	MD (95% CI)	*P*
Involved limb	8.1 ± 3.6	12.6 ± 2.6	4.5 (2.7-6.3)	**<.001**
Uninvolved limb	8.9 ± 3.4	12.9 ± 2.4	4.0 (2.3-5.7)	**<.001**

a Data are presented as mean ± SD unless otherwise indicated. Boldface *P* values indicate statistically significant difference between groups (*P* < .05). CI, confidence interval; MD, mean difference; PFI, patellofemoral instability.

The interrater reliability for both knees (n = 36) was good, with an ICC of 0.785 (95% CI, 0.579-0.890) and an MDC of 3.1 mm. The intrarater reliability for both knees (n = 8) was excellent, with an ICC of 0.958 (95% CI, 0.790-0.992) and an MDC of 1.7 mm (Table 3).

**Table 3:** Inter- and Intrarater Reliability of Measurements^*a*^

Variable	ICC (95% CI)	MDC_95_, mm	SEM
Interrater reliability			
Right side (n = 18)	0.794 (0.450-0.924)	3.08	1.11
Left side (n = 18)	0.796 (0.455-0.924)	3.02	1.09
Both sides (n = 36)	0.785 (0.579-0.890)	3.10	1.12
Intrarater reliability			
Right side (n = 4)	0.991 (0.869-0.999)	0.88	0.32
Left side (n = 4)	0.921 (0.217-0.995)	2.34	0.84
Both sides (n = 8)	0.958 (0.790-0.992)	1.71	0.62

a CI, confidence interval; ICC, intraclass correlation coefficient; MDC, minimal detectable change.

## Discussion

The novel US-based measurement of axial patellofemoral alignment described in this study demonstrated good-to-excellent reliability. When compared with controls, MPT-LTR distance was significantly smaller in the PFI group, indicating a more lateral position of the extensor mechanism and providing construct validity of this measure. US based-based measures offer the benefits of ease of use, increased access, and decreased cost compared with MRI scan. Leveraging US imaging in patients with PFI may allow for more efficient clinical care. In addition, the ease of access and efficiency of utilizing US would allow for serial measurements over time to evaluate their response to treatment as well the capacity for contralateral imaging or screening that may not be feasible with MRI scan.

The novel assessment described in this study takes advantage of the easily accessible soft tissue visualization of US and the inherent, closely associated relationship of the patella to the patellar tendon. The patellar tendon serves as the distal attachment of the quadriceps muscle group to the tibia and force transmitted through the patellofemoral joint by the quadriceps plays a key role in maintaining patellar stability. If the patellar tendon demonstrates a more lateral orientation relative to the trochlear ridge, this relationship may create a more lateral force vector upon quadriceps contraction, creating dynamic forces that may contribute to lateral patellar subluxation or dislocation.

In 2018, Mistovich et al^9^ reported on the reliability and discriminate validity of a novel measure axial patellar alignment utilizing MRI. In this latter study, the authors utilized various slices of axial plane MRI to determine the width of the patellar tendon that extended beyond the lateral trochlear ridge. This measurement is similar in concept to the US-based measure described in the current study. Mistovich et al^9^ theorized that a larger proportion of the patellar tendon uncontained by the trochlea would lead to more lateral force vector upon quadriceps contraction and subsequently increase the risk of patellar instability. In support of this theory, the results of their study demonstrated patients with instability had significantly more patellar tendon width outside the lateral trochlear ridge when compared with controls.

Previous work has described US-based measures to assess patellofemoral anatomy. Kwan et al^7^ performed a cadaver-based validation study of an US measure describing the distance between the lateral edge of the patella and the lateral femoral condyle. They reported a good correlation between US and direct measurement of lateral patellar positioning in this manner. Similarly, Bolgla et al^3^ assessed the relationship of the patella to the trochlea in a group of women with patellofemoral pain utilizing US. These authors utilized a modification of a measurement technique first described by Anillo et al^1^ in 2009 to quantify the relationship of the patella to the central aspect of the femoral trochlea. Although it was noted that those with patellofemoral pain had a more lateral patellar position than controls, this difference was not statistically significant.

Even though a lateral patellar position may be a shared characteristic between those with patellar instability and patellofemoral pain syndrome, the current study expanded upon this measurement technique to make it more specific to the mechanism of injury associated with instability. Specifically quantifying the relationship of the patellar tendon to the lateral trochlear ridge, rather than offset from the central aspect of the trochlear groove, allows for better identification of the “tipping” point or margins of stability for sustaining a patellar subluxation or dislocation event. In theory, it is not necessarily important to understand how centralized the patella is within the trochlear groove, but rather it is more important to understand how close the patella is to disengaging from the trochlea. The US measure utilized here directly quantifies that relationship and is consistent with findings outlining similar concept of patellar instability.^9^ Future research utilizing this US method should be conducted with a larger population of persons with patellar instability and seek to establish a cutoff value that may be associated with higher risk of initial or repeat dislocations. In addition, the dynamic nature of US can be leveraged to visualize the change in position that may occur during movement (ie, as the patella engages in the trochlea during flexion) and under muscular contraction (ie, degree of lateral translation during quadriceps contraction). Measures of this nature may offer more ecological validity in patellar instability injury dynamics and are currently being studied at our institution.

We found good to excellent intra- and interrater reliability with this measurement procedure. The mean difference in MPT-LTR distance between the PFI and control groups were 4.5 and 4.0 mm in the involved and uninvolved limbs, respectively, which exceeded the MDC for the measure (3.1 mm). This finding further bolsters the clinical relevance of our data, indicating the differences seen between populations was more than what can be accounted for by measurement variability.

Interestingly, there were no differences in the MPT-LTR distance between limbs in either the PFI or control groups. These results indicate that adolescents with a history of patellar instability demonstrate a more lateralized patella on both the involved and uninvolved limb. To this point, 4 out of 24 (16%) of the patients with PFI in this study had a history of bilateral patellar instability events. This finding may be congruent with previous research that has demonstrated a high risk of contralateral patellar dislocation, particularly among adolescents, after a primary dislocation event.^4,5,11^ The utility of US-based measures of patellar positioning offer significant potential for bilateral examination or serial monitoring to gain further insight into contralateral injury risk and changes in patellar position that may be associated with nonoperative or operative treatment. Future research should be directed toward longitudinal assessment of patellar positioning within this population and understanding how it relates to repeat patellar instability, contralateral instability, or whether nonoperative rehabilitation efforts may affect this measurement.

### Limitations

The current work is not without limitations. First, patients were recruited from the orthopaedic clinic, which may introduce selection bias, as the included patients with PFI in this setting may have more severe anatomy than the general population of patients with PFI. Intrarater reliability testing was completed only in a convenience sample of patients who returned to the clinic. The US-based assessment described in this study was novel, and all measures were performed by persons who helped in its development. Given the technique-dependent nature of musculoskeletal US, further work is necessary to evaluate the reproducibility of this measure in providers outside of the study team. Nevertheless, the 2 members of the study team who conducted the image acquisition and measurements (E.G. and M.B.) do not routinely use musculoskeletal sonography in their routine clinical practice. Thus, we feel that the imaging protocol can be highly generalizable to those with minimal ultrasonography experience. Lastly, patient-specific factors, such as body mass index, patellar height, or trochlea dysplasia, could have affected the reliability of this measurement; however, these factors were unable to be evaluated given the imaging and sample size of the studied cohort.

## Conclusion

The US-based MPT-LTR distance described in this study was found to represent a reliable method for assessing lateral patellar positioning and may be an efficient and cost-effective adjunct imaging modality for patellar instability. Participants with a history of PFI had a statistically significant difference in MPT-LTR measures, indicating a more laterally oriented extensor mechanism and differentiating them from the control group. Future research should focus on establishing relationships between the US-based MPT-LTR measure and existing radiographic or MRI-based measures, defining values of the US-based MPT-LTR indicative of the higher risk of recurrent instability, and refining this measure to add more dynamic visualization of patellofemoral mechanics.
